# Crystal structure of 4-methyl­sulfanyl-2-(2*H*-tetra­zol-2-yl)pyrimidine

**DOI:** 10.1107/S2056989015023634

**Published:** 2015-12-16

**Authors:** Andreas Thomann, Volker Huch, Rolf W. Hartmann

**Affiliations:** a Helmholtz-Institute for Pharmaceutical Research Saarland (HIPS), Department for Drug Design and Optimization (DDOP), Saarland University, Campus E8.1, D-66123 Saarbruecken, Germany; b Department of Inorganic Chemistry, Saarland University, Campus B2.2, D-66123 Saarbruecken, Germany; c Department of Pharmacy, Pharmaceutical and Medicinal Chemistry, Saarland University, Campus C2.3, D-66123 Saarbruecken, Germany

**Keywords:** crystal structure, tetra­zole, pyrimidine, thio, heterocyles, S_N_Ar reactions, π–π inter­actions

## Abstract

The title compound, C_6_H_6_N_6_S, crystallized with two independent mol­ecules (*A* and *B*) in the asymmetric unit. The conformation of the two mol­ecules differs slightly. While the tetra­zole ring is inclined to the pyrim­idene ring by 5.48 (7) and 4.24 (7)° in mol­ecules *A* and *B*, respectively, the N—C—S—C torsion angles of the thio­methyl groups differ by *ca* 180°. In the crystal, the *A* and *B* mol­ecules are linked *via* a C—H⋯N hydrogen bond. They stack along the *b*-axis direction forming columns within which there are weak π–π inter­actions present [shortest inter-centroid distance = 3.6933 (13) Å].

## Related literature   

For applications of tetra­zolyl-substituted aromatic systems in metal–ligand research, see: Kim *et al.* (2008 ▸); Stoessel *et al.* (2010 ▸); in drug development, see: Pasternak *et al.* (2012 ▸); Biswas *et al.* (2015 ▸); in polymer synthesis, see: Yu *et al.* (2008 ▸); Sengupta *et al.* (2010 ▸). For the synthesis of 4-methyl­sulfanyl-2-(1*H*-tetra­zol-1-yl)pyrimidine and the title compound, see: Thomann *et al.* (2014 ▸).
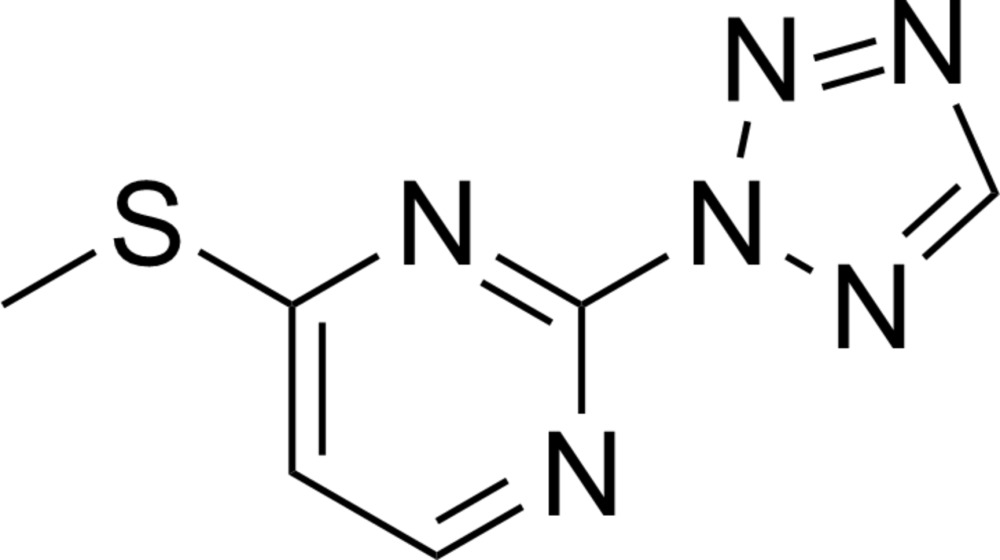


## Experimental   

### Crystal data  

C_6_H_6_N_6_S
*M* *_r_* = 194.23
Triclinic, 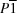

*a* = 6.3001 (17) Å
*b* = 7.393 (2) Å
*c* = 18.159 (5) Å
α = 91.407 (7)°
β = 95.864 (7)°
γ = 102.695 (8)°
*V* = 819.9 (4) Å^3^
*Z* = 4
Mo *K*α radiation
μ = 0.35 mm^−1^
*T* = 143 K
0.22 × 0.22 × 0.01 mm

### Data collection  

Bruker APEXII CCD diffractometer
Absorption correction: multi-scan (*SADABS*; Bruker, 2010 ▸) *T*_min_ = 0.716, *T*_max_ = 0.746
15501 measured reflections
4581 independent reflections
3596 reflections with *I* > 2σ(*I*)
*R*_int_ = 0.028

### Refinement  

*R*[*F* ^2^ > 2σ(*F* ^2^)] = 0.034
*wR*(*F* ^2^) = 0.086
*S* = 1.01
4581 reflections
283 parameters
All H-atom parameters refined
Δρ_max_ = 0.35 e Å^−3^
Δρ_min_ = −0.30 e Å^−3^

### 

Data collection: *APEX2* (Bruker, 2010 ▸); cell refinement: *SAINT* (Bruker, 2010 ▸); data reduction: *SAINT*; program(s) used to solve structure: *SHELXS97* (Sheldrick 2008 ▸); program(s) used to refine structure: *SHELXL2014* (Sheldrick, 2015 ▸); molecular graphics: *PLATON* (Spek, 2009 ▸); software used to prepare material for publication: *SHELXL2014* and *PLATON*.

## Supplementary Material

Crystal structure: contains datablock(s) I. DOI: 10.1107/S2056989015023634/su5253sup1.cif

Structure factors: contains datablock(s) I. DOI: 10.1107/S2056989015023634/su5253Isup2.hkl

Supporting information file. DOI: 10.1107/S2056989015023634/su5253Isup3.cml

**Fig. 1.**
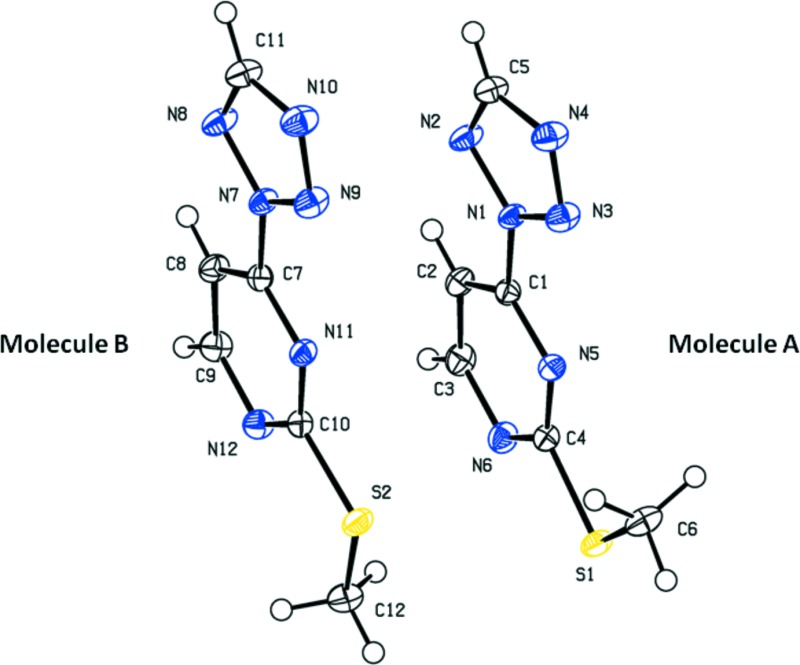
The molecular structure of the two independent molecules (A and B) of the title compound (2), with atom labelling. Displacement ellipsoids are drawn at the 50% probability level.

A B . DOI: 10.1107/S2056989015023634/su5253fig1.tif
The mol­ecular structure of the two independent mol­ecules (*A* and *B*) of the title compound (2), with atom labelling. Displacement ellipsoids are drawn at the 50% probability level.

**Fig. 2.**
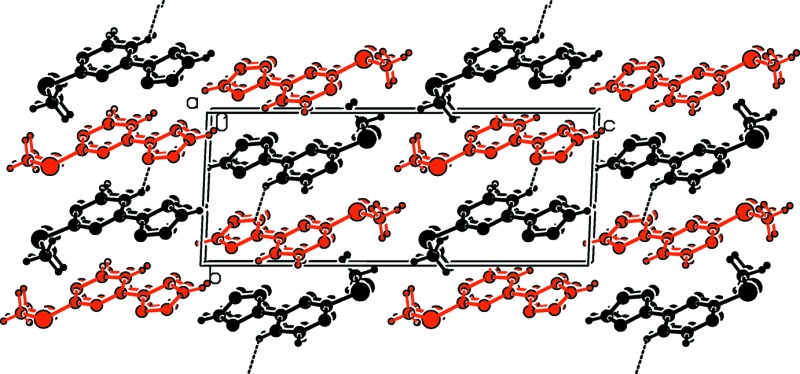
The crystal packing of the two independent molecules (A black; B red) of the title compound (2), viewed along the a axis. Hydrogen bonds are shown as dashed lines (see Table 1).

A B a . DOI: 10.1107/S2056989015023634/su5253fig2.tif
The crystal packing of the two independent mol­ecules (*A* black; *B* red) of the title compound (2), viewed along the *a* axis. Hydrogen bonds are shown as dashed lines (see Table 1).

**Fig. 3.**
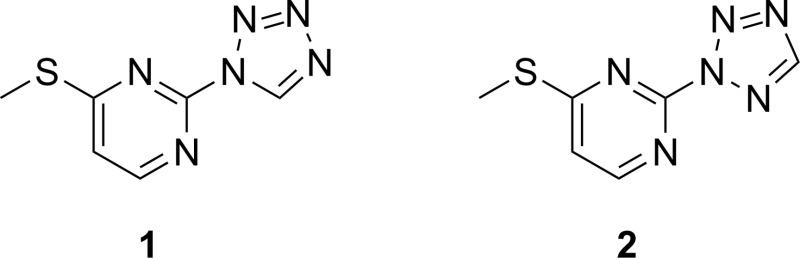
Compounds (1) and (2).

. DOI: 10.1107/S2056989015023634/su5253fig3.tif
Compounds (1) and (2).

CCDC reference: 1441424

Additional supporting information:  crystallographic information; 3D view; checkCIF report

## Figures and Tables

**Table 1:** Hydrogen-bond geometry (Å, °)

*D*—H⋯*A*	*D*—H	H⋯*A*	*D*⋯*A*	*D*—H⋯*A*
C2—H1⋯N9^i^	0.89 (2)	2.58 (2)	3.203 (2)	129 (2)

Symmetry code: (i) 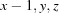.

## supplementary crystallographic information

### S1. Comment

4-tetra­zolyl­pyrimidines are well reported scaffolds in many bioactive entities. Besides synthetic chemistry, tetra­zolyl substituted aromatic systems are also of high inter­est for example, in metal-ligand research (Kim *et al.*, 2008; Stoessel *et al.*, 2010), drug development (Pasternak *et al.*, 2012; Biswas *et al.*, 2015) and polymer discovery (Yu *et al.*, 2008; Sengupta *et al.*, 2010). Thus, the knowledge of the three dimensional structure of these moieties is of crucial importance for the rational design in these fields of research. Recently, we have reported a novel method to synthesize such compounds (Thomann *et al.*, 2014). We have reported the synthesis of 4-(methyl­thio)-2-(1*H*-tetra­zol-1-yl)pyrimidine (1). Inter­estingly, when scaling up the reaction, another product was found in small amounts. NMR analytical characterization revealed the compound to be the 2-tetra­zolyl regioisomer (2). To determine unequivocally proof of the structure of this compound, we determined its crystal structure.

The title compound (2), crystallized with two independent molecules (A and B) in the asymmetric unit (Fig. 1). Inter­estingly, the two molecules differ in their conformation. While the tetra­zole moieties are arranged similarly, with the tetra­zole ring is inclined to the pyrim­idene ring by 5.48 (7) and 4.24 (7) ° in molecules A and B, respectively, the thio­methyl groups have a difference of the torsion angle about the C_ar_···S bond of *ca* 180° [for example, torsion angle N5—C4—S1—C6 = 0.89 (12) °, compared to torsion angle N11—C10—S2—C12 = −176.78 (10) °] indicating higher rotational freedom than the tetra­zoles (Fig. 1). The latter finding is of importance for computational chemists in medicinal chemistry, as the polarized hydrogen at atom C5 of the tetra­zole ring is able to form non-classical hydrogen bonds. Therefore, the results from the crystal structure may favour this conformational isomer for *in silico* predictions.

In the crystal, the A and B molecules are linked *via* a C—H···N hydrogen bond (Table 1 and Fig. 2). They stack along the *b* axis direction forming columns within which there are weak π-π inter­actions present [shortest inter-centroid distance is *Cg*2···*Cg*4^i^ = 3.6918 (5) Å; *Cg*2 and *Cg*4 are the centroids of rings N5/N6/C1—C4 and N11/N12/C7—C10, respectively; symmetry code: (i) *x*, *y* + 1, *z*].

### S2. Synthesis and crystallization

The title compound (2), was synthesized following a previously reported procedure (Thomann *et al.*, 2014). A mixture of 4-chloro-2-(methyl­thio)­pyrimidine, 1*H*-tetra­zole and tri­ethyl­amine, in the ratio 1:1:1, was stirred under microwave irradiation at 50 W, 353 K for 1 h. The crude product was purified by flash chromatography (hexane:ethyl acetate, 8:2, *R*_f_ = 1/4) to yield a white solid (9%). Crystals formed at 294 K after 16 h from a saturated solution of 2 in ethyl acetate.^1^H NMR (CDCl_3_, 300 MHz) 8.80 (dd, *J* = 5.3, 0.6 Hz, 1 H), 8.77 (s, 1 H), 7.77 (dd, *J* = 5.3, 0.7 Hz, 1 H), 2.69 p.p.m. (d, *J* = 0.7 Hz, 3 H).

### S3. Refinement

Crystal data, data collection and structure refinement details are summarized in Table 2. H atoms were located in a difference Fourier map and freely refined.

### Crystal data

**Table:**

C_6_H_6_N_6_S	*Z* = 4
*M**_r_* = 194.23	*F*(000) = 400
Triclinic, *P*1	*D*_x_ = 1.574 Mg m^−^^3^
*a* = 6.3001 (17) Å	Mo *K*α radiation, λ = 0.71073 Å
*b* = 7.393 (2) Å	Cell parameters from 728 reflections
*c* = 18.159 (5) Å	θ = 3.6–24.3°
α = 91.407 (7)°	µ = 0.35 mm^−^^1^
β = 95.864 (7)°	*T* = 143 K
γ = 102.695 (8)°	Cuboid, colourless
*V* = 819.9 (4) Å^3^	0.22 × 0.22 × 0.01 mm

### Data collection

**Table:**

Bruker APEXII CCD diffractometer	3596 reflections with *I* > 2σ(*I*)
φ and ω scans	*R*_int_ = 0.028
Absorption correction: multi-scan (*SADABS*; Bruker, 2010)	θ_max_ = 29.6°, θ_min_ = 2.3°
*T*_min_ = 0.716, *T*_max_ = 0.746	*h* = −8→8
15501 measured reflections	*k* = −10→10
4581 independent reflections	*l* = −24→25

### Refinement

**Table:**

Refinement on *F*^2^	0 restraints
Least-squares matrix: full	Hydrogen site location: difference Fourier map
*R*[*F*^2^ > 2σ(*F*^2^)] = 0.034	All H-atom parameters refined
*wR*(*F*^2^) = 0.086	*w* = 1/[σ^2^(*F*_o_^2^) + (0.0376*P*)^2^ + 0.2718*P*] where *P* = (*F*_o_^2^ + 2*F*_c_^2^)/3
*S* = 1.01	(Δ/σ)_max_ = 0.001
4581 reflections	Δρ_max_ = 0.35 e Å^−^^3^
283 parameters	Δρ_min_ = −0.30 e Å^−^^3^

### Special details

**Table:**

Geometry. All e.s.d.'s (except the e.s.d. in the dihedral angle between two l.s. planes) are estimated using the full covariance matrix. The cell e.s.d.'s are taken into account individually in the estimation of e.s.d.'s in distances, angles and torsion angles; correlations between e.s.d.'s in cell parameters are only used when they are defined by crystal symmetry. An approximate (isotropic) treatment of cell e.s.d.'s is used for estimating e.s.d.'s involving l.s. planes.

### Fractional atomic coordinates and isotropic or equivalent isotropic displacement parameters (Å^2^)

**Table:**

	*x*	*y*	*z*	*U*_iso_*/*U*_eq_	
S1	0.84117 (6)	0.83334 (5)	0.58281 (2)	0.01975 (9)	
N1	1.04303 (18)	0.69484 (15)	0.84617 (6)	0.0152 (2)	
N2	1.00906 (19)	0.60999 (17)	0.90974 (6)	0.0208 (3)	
N3	1.24517 (19)	0.79683 (17)	0.84553 (7)	0.0209 (3)	
N4	1.3498 (2)	0.77865 (18)	0.91059 (7)	0.0233 (3)	
N5	0.93885 (18)	0.76012 (15)	0.72442 (6)	0.0159 (2)	
N6	0.56784 (19)	0.65519 (16)	0.66803 (7)	0.0200 (3)	
C1	0.8749 (2)	0.67900 (17)	0.78527 (7)	0.0148 (3)	
C2	0.6647 (2)	0.58341 (19)	0.79335 (8)	0.0184 (3)	
C3	0.5146 (2)	0.5770 (2)	0.73096 (8)	0.0203 (3)	
C4	0.7801 (2)	0.74205 (18)	0.66783 (8)	0.0162 (3)	
C5	1.2034 (2)	0.6651 (2)	0.94818 (8)	0.0209 (3)	
C6	1.1309 (2)	0.9298 (2)	0.60068 (9)	0.0230 (3)	
H1	0.629 (3)	0.532 (2)	0.8352 (10)	0.028 (5)*	
H2	0.366 (3)	0.512 (2)	0.7313 (9)	0.023 (4)*	
H3	1.235 (3)	0.634 (2)	0.9944 (11)	0.030 (5)*	
H4	1.204 (3)	0.833 (2)	0.6178 (10)	0.032 (5)*	
H5	1.160 (3)	1.037 (2)	0.6366 (10)	0.030 (5)*	
H6	1.172 (3)	0.969 (3)	0.5531 (11)	0.040 (5)*	
S2	0.91337 (6)	0.35407 (5)	0.59998 (2)	0.02003 (10)	
N7	1.07557 (18)	0.19569 (15)	0.85809 (6)	0.0156 (2)	
N8	1.0376 (2)	0.11547 (17)	0.92240 (7)	0.0219 (3)	
N9	1.28032 (19)	0.29223 (17)	0.85739 (7)	0.0218 (3)	
N10	1.3817 (2)	0.27602 (18)	0.92304 (7)	0.0241 (3)	
N11	0.97742 (18)	0.26119 (15)	0.73636 (6)	0.0159 (2)	
N12	0.60819 (19)	0.16413 (16)	0.67866 (7)	0.0192 (2)	
C7	0.9101 (2)	0.18161 (17)	0.79690 (7)	0.0146 (3)	
C8	0.6975 (2)	0.08981 (19)	0.80433 (8)	0.0181 (3)	
C9	0.5511 (2)	0.0862 (2)	0.74162 (8)	0.0204 (3)	
C10	0.8194 (2)	0.24779 (18)	0.67912 (8)	0.0162 (3)	
C11	1.2311 (2)	0.1686 (2)	0.96118 (8)	0.0218 (3)	
C12	0.6629 (3)	0.3216 (2)	0.53901 (9)	0.0256 (3)	
H7	0.658 (3)	0.041 (2)	0.8462 (10)	0.028 (5)*	
H8	0.400 (3)	0.022 (2)	0.7410 (10)	0.027 (4)*	
H9	1.264 (3)	0.138 (3)	1.0104 (11)	0.035 (5)*	
H10	0.564 (3)	0.385 (2)	0.5600 (10)	0.035 (5)*	
H11	0.702 (3)	0.373 (3)	0.4949 (11)	0.037 (5)*	
H12	0.600 (3)	0.192 (3)	0.5294 (10)	0.038 (5)*	

### Atomic displacement parameters (Å^2^)

**Table:**

	*U*^11^	*U*^22^	*U*^33^	*U*^12^	*U*^13^	*U*^23^
S1	0.02103 (18)	0.02210 (18)	0.01490 (18)	0.00253 (13)	−0.00004 (13)	0.00614 (13)
N1	0.0152 (5)	0.0167 (5)	0.0130 (5)	0.0007 (4)	0.0036 (4)	0.0040 (4)
N2	0.0216 (6)	0.0250 (6)	0.0152 (6)	0.0020 (5)	0.0040 (5)	0.0078 (5)
N3	0.0155 (6)	0.0260 (6)	0.0190 (6)	−0.0011 (5)	0.0024 (5)	0.0049 (5)
N4	0.0203 (6)	0.0295 (7)	0.0186 (6)	0.0031 (5)	0.0005 (5)	0.0046 (5)
N5	0.0166 (5)	0.0159 (5)	0.0149 (6)	0.0028 (4)	0.0030 (4)	0.0019 (4)
N6	0.0182 (6)	0.0193 (6)	0.0212 (6)	0.0019 (4)	0.0009 (5)	0.0034 (5)
C1	0.0155 (6)	0.0138 (6)	0.0150 (6)	0.0030 (5)	0.0025 (5)	0.0011 (5)
C2	0.0186 (7)	0.0180 (7)	0.0181 (7)	0.0012 (5)	0.0057 (5)	0.0042 (5)
C3	0.0168 (7)	0.0200 (7)	0.0232 (8)	0.0008 (5)	0.0039 (6)	0.0026 (5)
C4	0.0181 (6)	0.0149 (6)	0.0157 (7)	0.0034 (5)	0.0026 (5)	0.0020 (5)
C5	0.0221 (7)	0.0251 (7)	0.0153 (7)	0.0044 (6)	0.0022 (6)	0.0045 (5)
C6	0.0203 (7)	0.0305 (8)	0.0190 (7)	0.0053 (6)	0.0042 (6)	0.0093 (6)
S2	0.02219 (18)	0.02074 (18)	0.01648 (18)	0.00230 (13)	0.00321 (14)	0.00600 (13)
N7	0.0134 (5)	0.0181 (5)	0.0149 (6)	0.0015 (4)	0.0037 (4)	0.0045 (4)
N8	0.0203 (6)	0.0281 (7)	0.0170 (6)	0.0028 (5)	0.0044 (5)	0.0090 (5)
N9	0.0152 (6)	0.0265 (6)	0.0213 (6)	−0.0005 (5)	0.0014 (5)	0.0057 (5)
N10	0.0182 (6)	0.0316 (7)	0.0203 (7)	0.0015 (5)	−0.0004 (5)	0.0048 (5)
N11	0.0159 (5)	0.0160 (5)	0.0155 (6)	0.0021 (4)	0.0029 (4)	0.0033 (4)
N12	0.0170 (6)	0.0214 (6)	0.0184 (6)	0.0020 (5)	0.0026 (5)	0.0016 (5)
C7	0.0139 (6)	0.0142 (6)	0.0160 (6)	0.0039 (5)	0.0019 (5)	0.0008 (5)
C8	0.0171 (7)	0.0200 (7)	0.0170 (7)	0.0018 (5)	0.0056 (5)	0.0036 (5)
C9	0.0164 (7)	0.0233 (7)	0.0206 (7)	0.0019 (5)	0.0034 (6)	0.0014 (5)
C10	0.0189 (7)	0.0139 (6)	0.0160 (7)	0.0039 (5)	0.0023 (5)	0.0008 (5)
C11	0.0188 (7)	0.0294 (8)	0.0168 (7)	0.0039 (6)	0.0023 (6)	0.0060 (6)
C12	0.0305 (8)	0.0281 (8)	0.0181 (8)	0.0075 (7)	−0.0006 (6)	0.0034 (6)

### Geometric parameters (Å, º)

**Table:**

S1—C4	1.7453 (15)	S2—C10	1.7487 (15)
S1—C6	1.8004 (16)	S2—C12	1.7992 (16)
N1—N3	1.3311 (16)	N7—N9	1.3314 (16)
N1—N2	1.3412 (16)	N7—N8	1.3421 (16)
N1—C1	1.4347 (17)	N7—C7	1.4291 (17)
N2—C5	1.3207 (19)	N8—C11	1.3182 (19)
N3—N4	1.3176 (17)	N9—N10	1.3148 (17)
N4—C5	1.356 (2)	N10—C11	1.3566 (19)
N5—C1	1.3267 (17)	N11—C7	1.3237 (17)
N5—C4	1.3412 (17)	N11—C10	1.3496 (17)
N6—C3	1.3336 (19)	N12—C10	1.3377 (18)
N6—C4	1.3506 (18)	N12—C9	1.3393 (19)
C1—C2	1.3815 (19)	C7—C8	1.3837 (19)
C2—C3	1.393 (2)	C8—C9	1.386 (2)
C2—H1	0.885 (19)	C8—H7	0.886 (19)
C3—H2	0.951 (17)	C9—H8	0.965 (18)
C5—H3	0.891 (19)	C11—H9	0.940 (19)
C6—H4	0.974 (17)	C12—H10	0.955 (19)
C6—H5	0.988 (17)	C12—H11	0.93 (2)
C6—H6	0.96 (2)	C12—H12	0.957 (19)
			
C4—S1—C6	101.92 (7)	C10—S2—C12	101.37 (8)
N3—N1—N2	113.78 (11)	N9—N7—N8	113.63 (11)
N3—N1—C1	123.45 (11)	N9—N7—C7	123.40 (11)
N2—N1—C1	122.75 (11)	N8—N7—C7	122.96 (11)
C5—N2—N1	101.28 (11)	C11—N8—N7	101.34 (12)
N4—N3—N1	105.82 (12)	N10—N9—N7	105.89 (12)
N3—N4—C5	106.17 (12)	N9—N10—C11	106.19 (12)
C1—N5—C4	114.47 (12)	C7—N11—C10	114.67 (12)
C3—N6—C4	115.71 (12)	C10—N12—C9	115.82 (12)
N5—C1—C2	125.31 (12)	N11—C7—C8	125.20 (12)
N5—C1—N1	115.33 (12)	N11—C7—N7	115.25 (12)
C2—C1—N1	119.36 (12)	C8—C7—N7	119.55 (12)
C1—C2—C3	114.59 (13)	C7—C8—C9	114.40 (13)
C1—C2—H1	122.4 (11)	C7—C8—H7	122.5 (11)
C3—C2—H1	123.0 (12)	C9—C8—H7	123.1 (12)
N6—C3—C2	123.19 (13)	N12—C9—C8	123.46 (14)
N6—C3—H2	116.5 (10)	N12—C9—H8	116.1 (11)
C2—C3—H2	120.3 (10)	C8—C9—H8	120.5 (11)
N5—C4—N6	126.72 (13)	N12—C10—N11	126.44 (13)
N5—C4—S1	119.87 (10)	N12—C10—S2	119.96 (10)
N6—C4—S1	113.41 (10)	N11—C10—S2	113.60 (10)
N2—C5—N4	112.96 (13)	N8—C11—N10	112.94 (13)
N2—C5—H3	124.0 (12)	N8—C11—H9	124.6 (12)
N4—C5—H3	123.0 (12)	N10—C11—H9	122.5 (12)
S1—C6—H4	108.9 (11)	S2—C12—H10	109.7 (11)
S1—C6—H5	110.3 (10)	S2—C12—H11	105.7 (12)
H4—C6—H5	112.5 (14)	H10—C12—H11	110.6 (16)
S1—C6—H6	103.5 (11)	S2—C12—H12	110.3 (11)
H4—C6—H6	110.8 (15)	H10—C12—H12	111.9 (16)
H5—C6—H6	110.4 (15)	H11—C12—H12	108.4 (16)

### Hydrogen-bond geometry (Å, º)

**Table:**

*D*—H···*A*	*D*—H	H···*A*	*D*···*A*	*D*—H···*A*
C2—H1···N9^i^	0.89 (2)	2.58 (2)	3.203 (2)	129 (2)

Symmetry code: (i) *x*−1, *y*, *z*.
