# Supplementary figures and images for: Crystal structure of 4-methyl­sulfanyl-2-(2H-tetra­zol-2-yl)pyrimidine
Source: Acta Crystallogr E Crystallogr Commun. 2015 Dec 16;71(Pt 12):o1051–2. doi: 10.1107/S2056989015023634 (PMC4719974; doi:10.1107/S2056989015023634)

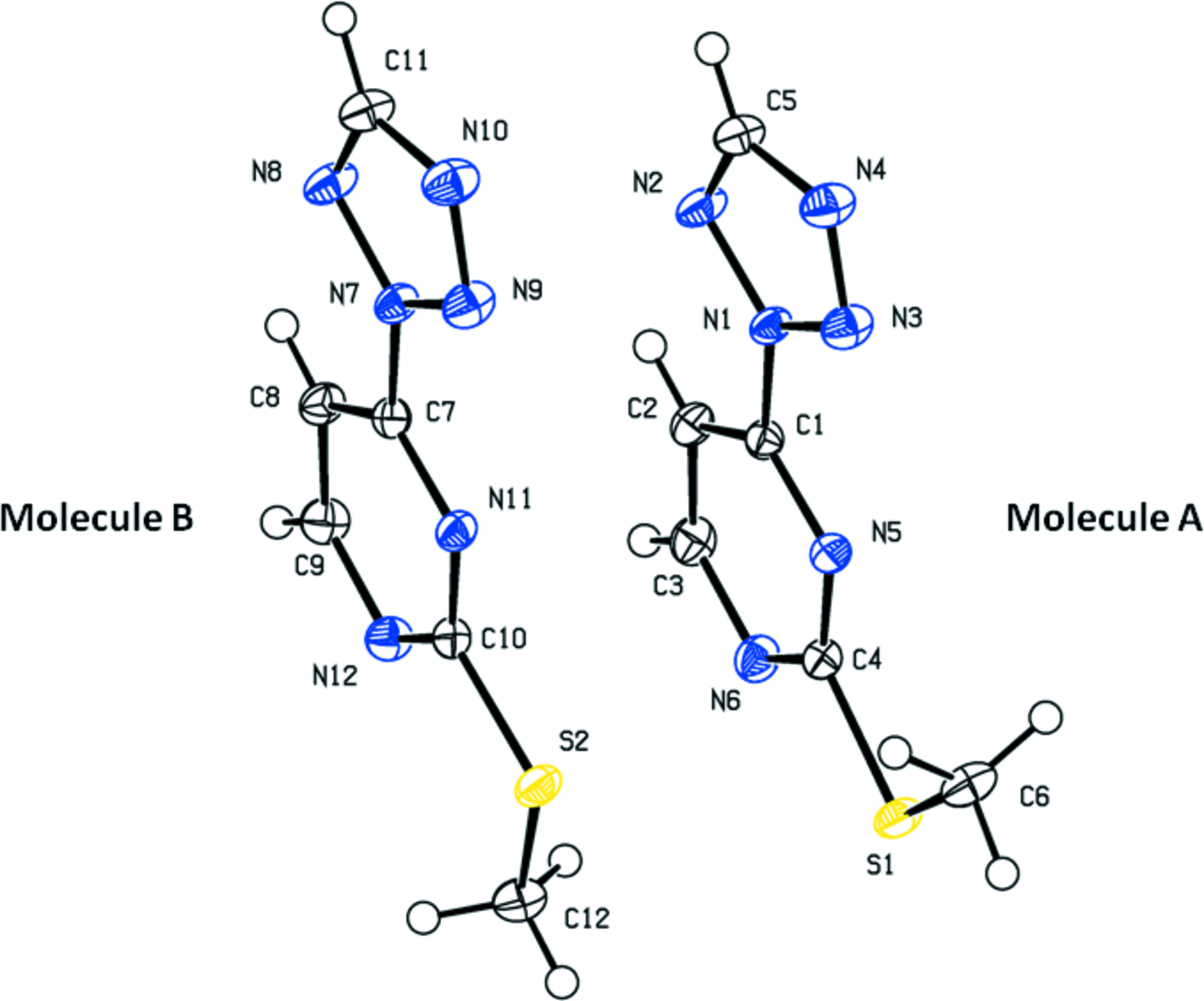

Supplement: Supplementary file 4 [file e-71-o1051-fig1.tif]

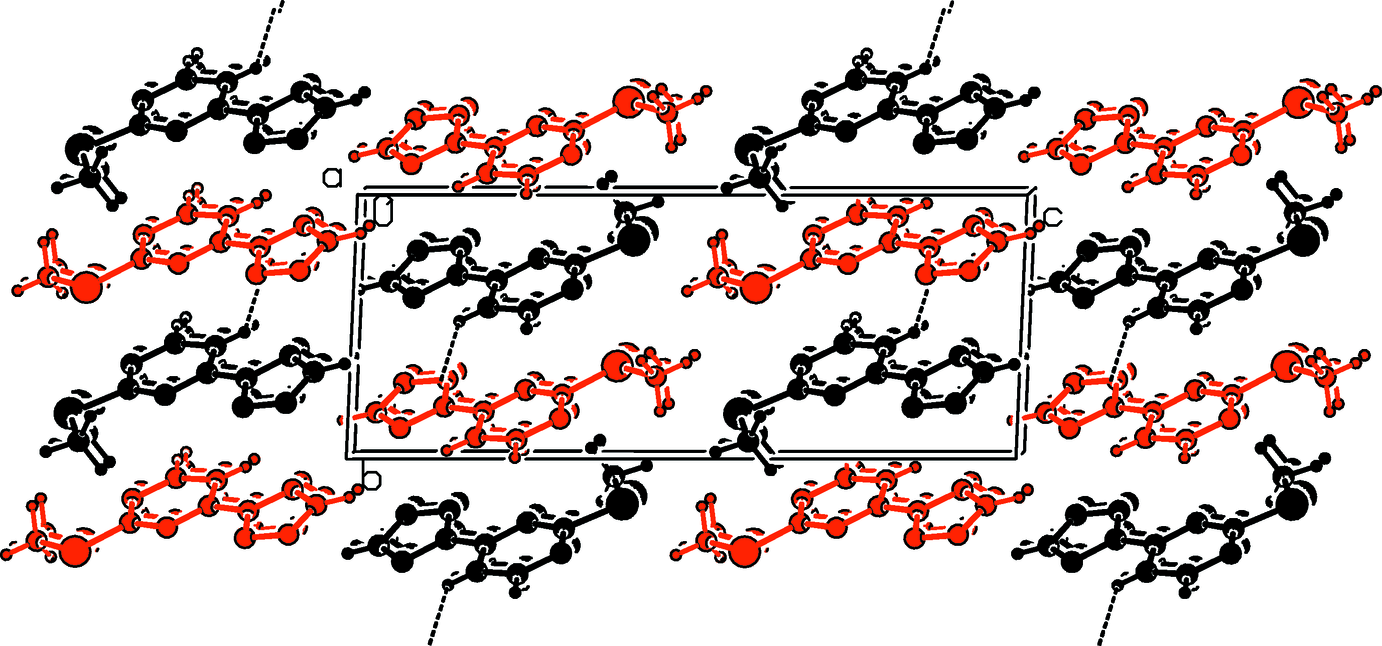

Supplement: Supplementary file 5 [file e-71-o1051-fig2.tif]

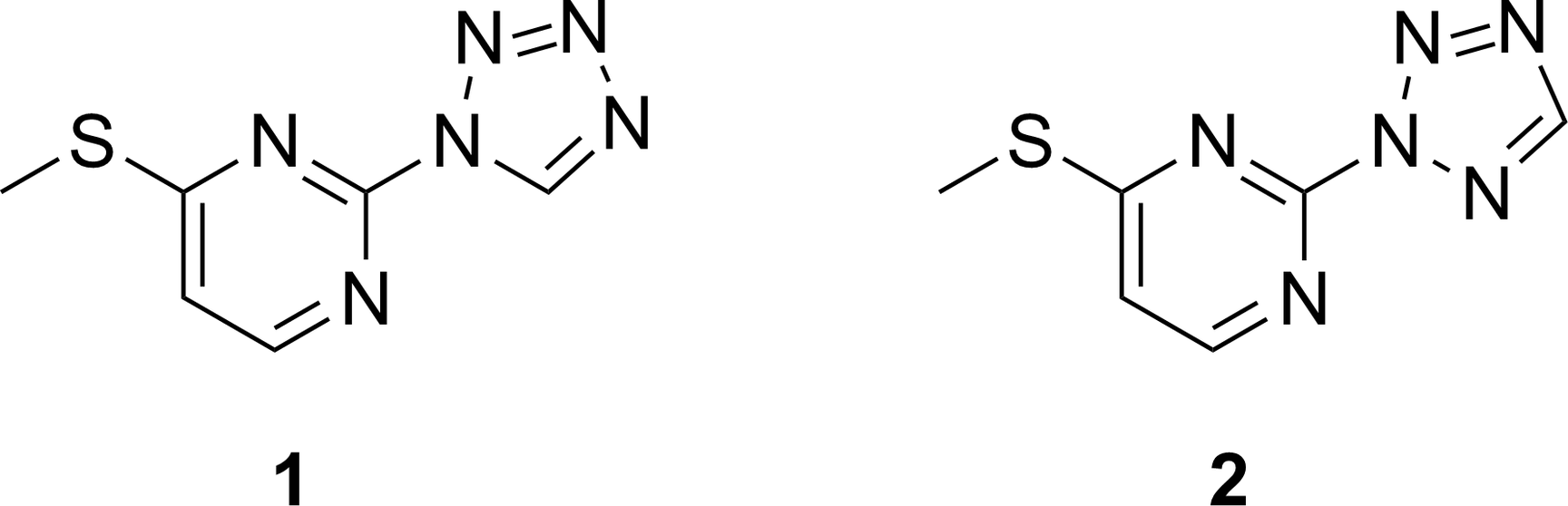

Supplement: Supplementary file 6 [file e-71-o1051-fig3.tif]
